# Control of Patients

**Published:** 1913-10-15

**Authors:** Wm. C. Gillespie

**Affiliations:** Nashville, Tenn.


### CONTROL OF PATIENTS.

BY WM. C. GILLESPIE, D.D.S., NASHVILLE, TENN.

Read before the Tennessee State Dental Association, June 6, 1913.

The proper control of patients in the dental chair is a subject of vital importance to every practicing dentist, and yet it is one never referred to in any college lecture the essayist ever heard, nor is it more than hinted at in Dental Association deliberations.

About 95 per cent of dentists in practice exercise little or no control whatever over their patients during operations, but treat them in the same manner, or with less thoughtfulness, than a fool parent does a badly spoiled child. The average dentist not only does not control his patient, but encourages misbehavior on his part.

Like the foolish parent who permits and encourages a child possessing enough self-will to do so to become an intolerable nuisance to itself and every one else, the average dentist ruins every patient who comes to him who is inclined to take advantage of the opportunity so unresistingly granted.

Again paralleling the foolish parent, all that is necessary to prevent the spoiling of a patient, or to reform a spoiled one, is the excercise of a little firmness and plain common sense.

To get directly to the subject under discussion we will make this statement, and defend the position against any adverse criticism. A tooth in any given condition is no more sensitive in the mouth of one person than one under the same conditions in another mouth where patients are in approximately the same condition.

The difference in conduct in the chair comes from difference in method of expression or manifestation of pain experienced rather than from difference in degree of pain actually experienced, and there should consequently be no marked difference in conduct of patients where the dentist controls himself and excercises the power of control easily at his command through the simple expedients of common sense employed in making statements to patients, unmotional firmness of manner and tone—avoiding abruptness—low, even tone of voice, and careful, correct handling of instruments during operations.

The first factor in spoiling patients is nervous eagerness to secure the commendations of the patient, which gives the patient a large opportunity to think he is bossing the job.

The next and largely influencing element is the silly and often times idiotic statements made and questions asked by the dentist. But few dentists willingly permit another dentist to hear him talk to his patients in the chair, and yet these same dentists can talk very intelligently outside of their operating rooms.

Fear that the patient will take offense and go somewhere else if not allowed to squirm and twist and groan and jump and grab the instruments, makes the majority of dentists permit their patients to go the limit from a whisper to a war-whoop, and from the twitching of a muscle to an aggravated case of St. Vitus' dance, even when the operator knows it is but minor pain he is inflicting.

Instead of exercising some semblance of control, the average operator sweats, swears internally and sweetly and deliberately lies to his patient by saying "You have the most sensitive teeth I ever saw!" And he lies to the next patient in exactly the same words and soon gets his constitution so saturated with it, he says it mechanically without ever exercising a single brain-cell to do it.

When the patient is told this, vanity comes to the front and a continous performance of high grade contortion exhibitions and a volley of all the superlative adjectives in the English language is brought into play to demonstrate just how very much more sensitive those teeth are than any since the creation of the first set of teeth.

The patient feels that the good name and reputation of those teeth are at stake and a record for sensitiveness must be preserved if it is necessary to break a leg or rupture a lung to do it.

When a patient flinches the dentist with a flash of brilliant intellectual inspiration asks "Did that hurt you?"

The patient is thereby profoundly impressed with the fact that the dentist has absolutely no means of knowing whether or not he inflicts pain except from a heart-rending groan or a muscular convulsion.

Realizing from thence on the dentist's monumental ignorance of the tissues and structures upon which he is working and that he is liable at any instance to mortally injure them unless they head him off, they never again let him go so far as to really hurt again.

A large number of dentists instruct patients to raise a handor give some other sign when the instrument causes pain. Right there is laid the foundation for the purest, most unadulterated fake performances that could well be perpetrated, and the patient will often feel that a real kindness has been done the poor ignorant dentist who does'nt know what he is doing.

Again may the good Lord deliver us and our patients from the "funny" dentist, who, at any manifestation of pain bubbles over with mirth and perpetrates alleged witicisms that had been worn slick and shiny at the dawn of the stone age.

Again let us pray for the passing of the loquacious dentist who with fiendish persistance discusses endless topics while the patient's mouth is full of instruments and rubber dam, and doesn't care any kind of a dam for what is being said, or if interested, is in no frame of mind to listen or be able to reply.

Let us pray for the quiet passing of these varieties of dentists rather that they should get what they deserve.

No intelligent dentist can expect a patient to become quiet and tractable when subjected to such treatment, and no patient can become so who is not possessed of a supernormal power of self-repression.

Now just a few suggestions as to how control may be secured.

When the patient is in the chair the dentist should always speak in a low, even tone of voice.

If the patient is of nervous temperament or shows indications of nervou ness, or gives indication of being a patient who will be troublesome to operate for, tilt the chair back until they are forced to look you in the eye while you talk and in a quiet, kindly but firm way give them to understand that they have got to do their part in bearing whatever pain it becomes necessary for you to inflict. Give them to understand you know when you are causing pain and when you are not and that you and not themselves will shield them from unnecessary pain.

Tell them you can not and will not work unless they control themselves and make an honest effort to keep still. Tell them all that is necessary to be told, with the chair tilted back so they must look you directly in the eye and hold their eye during all you have to say. If they are sitting up their attention is distracted by every object in sight and the force of suggestion is entirely lost.

Talk as intelligently to your patients about their teeth as you can and would do about any other subject.

Never intimate to or agree with a patient that they are in the least differently constituted from any other human being. Even if you recognize some truth in what they say don't be fool enough to admit it to them. Of course that does not mean not to recognize any real physiological or constitutional abnormality, but never encourage a patient in the idea that he or she is any such freak as they are priding themselves on being.

Instead of creating or encouraging the mental suggestion that they are different from ordinary beings and thereby making trouble for yourself, impress upon them that they in no wise differ from other human beings and thereby reap the benefit to come of it through an attempt on their part to show you they are like other beings.

The psychology of this is indesputable. Do not irritate and lessen your patients ability to undergo the ordeal of the dental chair by an infernal stream of talk. The least said at the chair beyond the actual information necessary to be imparted, the greater ease with which you will control your patient.

Be reasonably pleasant at all times and talk all you please before and after the operation, but for the sake of humanity and the intelligent status of the dental profession cut out all the senseless old Methusela jokes around the chair.

The finest rule in the world to go by on the talking proposition is "When the patient's mouth is open, keep your own mouth shut."

Use scrupulous care not to unnecessarily inflict pain upon your patients, and then when they tell you they can't help making extreme manifestations of pain, don't hesitate to tell them they *can* do so, and insist that they do it, and most of your troubles will vanish.

DISCUSSION.

By Dr. Jas. A. Winn.—I think the essayist advanced some ideas that are good for us to think about, and we shall do well to follow some of his advice. If we expect to be successful as dental practitioners, we must certainly be the dentists, and have our patients be patient, and insist on their doing as we suggest. I think all that is necessary; in most cases I have found it so—is to assume a positive tone in speaking with patients, and there will be little trouble in controlling them. Of course we sometimes have an exceptionally nervous patient, in such cases usually all that is necessary is to assure them that we will be as careful as possible, and ask them to leave it to us and they will come out all right.

I don't mean to say, however, that we can always control them satisfactorily. I heard of a patient today that I

fear Dr. Gillespie did not successfully manage as he had to send for another dentist to give him gas, so that he could do an operation for him. He did not know I was going to hear about that, or tell it, but it came to my ears this afternoon, and I thought this would be a good time to tell it on him. I am sure all of us do get patients occasionally that are hard to manage. Patients are of different temperaments, and some of them when they come to us do not intend we shall do anything more for them than they can possibly get out of. Sometimes they are sure we are going to almost kill them; when I find one like that I usually suggest to them that if they expect me to treat their teeth, I shall expect them to sit still, and do as I say, and usually a little positive talk of that kind will bring them around alright.

I think this subject well worth consideration and discussion, and I hope the rest of you will enlarge on some of Dr. Gillespie's ideas and give us your methods.

By Dr. A. J. Cottrell.—As practitioners of dentristry, we have neglected a very great opportunity because we have not taken into consideration the psychological phases concerned in controlling patients. We are prone to make light of the theories of the New Thought people, and the ideas of Christian Scientists, and our good women that take up with these fads, and accept them, but, do you know, we are missing a great deal of comfort that we might have in this life if we would pay a little more attention to these things.

There is one phase that the New Thought people refer to often, and that is the "slavery of fear," or bondage that we find ourselves in through life, simply because we are afraid of what is going to happen—not the ills that we have, but those we are afraid are going to happen. That is the condition of the average patient that comes to our office, and Dr. Gillespie is only following out well established psychological principles in the course he endorses.

The New Thought people will tell you that thoughts are things, actual entities, that they are once given birth for all time to go radiating and radiating through space and time and eternity, actually exerting an influence for all time, and upon every one with whom they come in contact. Now I don't pretend to state this as a fact, but it is one of their theories, and a very comfortable one. If it is your attitude and your belief it will influence your feelings and direct your behavior when you come to deal with your patients, or it may be reflected from the patient.

Have you ever noticed the old-fashioned country doctor when he comes in to see the sick child? He takes up the little fellow's hand and notes his pulse, he lays it down and walks off. He has not said a word, and yet nine times out of ten he has condemned that child.

Dr. Gillespie has only suggested the carrying out of the things that have been established. We have overlooked these things, and I hope we will take Dr. Gillespie's suggestions to heart, and think about and make practical application as opportunity offers. The possibilities are unlimited, and I desire to congratulate Dr. Gillespie on being the first person that has ever brought this before a dental body in an intelligent way.

Dr. C. E. Hines.—This is one of the best papers I ever listened to. I try to do all my talking the first time I have the patient in my chair. I do as little work as possible at that time and I try to do all my talking then.

In a way, we all have to tell stories and we have to deceive our patients at times. We can not get around it.

I had a dream the other night. I dreamed that I had died and had gone up to the gate of heaven. There I saw a ladder extending away up into the skies, and there was a blackboard as far as eye could see by the side of that ladder. St. Peter handed me a piece of chalk, and says to me, "Hines, start up that ladder, and for every lie and misrepresentation that you have made to your patients make a mark as you go up." I went up a considerable distance, doing very nicely with my chalk, and I met my friend Dr. Gillespie coming back down. I said, "Hello, Gillespie, what is the matter?" He said, "I am out of chalk."

Dr. Jordan: I could not but be impressed by one of Dr. Gillespie's first statements, where he said, as I understood it, that, practically, the mouth of every patient was susceptible to the same degree of pain. If this is the opinion of this body of dentists I am very much mistaken in my own interpretation of the pain I witness under my operations.

Dr. Gillispie: You misunderstood me in that, Doctor. The idea I intended to convey there is that this trouble comes, not from one patient suffering so much more than another; that taking two of approximately the same condition of the teeth to be operated on, one patient will make an exaggerated statement of what he suffers and the other will minimize it, when they both suffer probably the same, and that there is where the control effort must be used, to hold both down to a minimum expression.

Dr. Jordan resumes —I think this is where Dr. Gillespie is absolutely and positively mistaken, for the simple reason if this patient misrepresents his suffering, the mind is controlling, and he is suffering just as much in his mind in this pain as he represents it to be. I do not deny but what you can control that to a certain extent, and work him down to where he will behave much better. You can do it and I have done it often. Just tell him to sit as still as he can and "holler" as loud as he pleases, as it is all right while you go right on.

I never failed to get in a filling once started, or to take out a nerve when practicable by any means. I took out yesterday four live, healthy, kicking pulps for one young man, and he had his head hanging back of the chair, but he didn't "holler," but he did perspire a little. He said he thought he was going to die, but that it didn't hurt so much. He says, "I'll come back tomorrow. Don't let up until we get them all out." I have got to take out about eight or ten more pulps for that boy, yet. He won't have more than six or eight live pulps in his teeth. He suffered ten times more when he first came to the office than he has suffered since.

I have patients I could take out live pulps for and not use cocain, not use anything. Such patients are more cold-blooded than other patients. They can stand more, or they do not feel the pain as others do. But with a nervous patient not under control, you can't touch the pulp. You will find that this type of patient will suffer more than others, and you can not control them all, any more than you can children. By the same method—one you can control by kindness, and another one you can control by looking him in the eye, but some you can't control by any means

Dr. Bates: Dr. Gillespie has presented a very interesting phase of the practice of dentistry, and to illustrate his point of the control of the patients, I will state an instance that occurred with me the other day, when I had to remove a live pulp. Although it had been partially anesthetized with cocain, when I removed it he felt pain. He said, "Let's see those instruments." I showed it to him. He said, "Doctor, I bet hell is full of those things." I said to the patient, "No, heaven is full of these things. They are to relieve suffering and relieve pain. That pain you experienced was momentary, but if that pulp had not been removed you would have had a continous pain."

Munsterburg has demonstrated scientifically, beyond a doubt, that suggestion plays the greatest part of all, that suggestion will control the mind of every man. He has shown that every man is himself a hypnotist, in that, by the proper suggestion, he is able to control to a certain extent—some, more than others. The looking in the eye is no more than the suggestion, and only by suggestion are we able to control.

As to whether we are permitted to deceive the patient, that depends largely on the characteristics of the patient. We must undoubtedly take into consideration the characteristics of the patient in order that we may control that patient. With one you must be decided and determined, with another you must be pleading, or kind and persuasive, but always in the control of the patient it is suggestion that produces the control.

Dr. Henry W. Morgan: The control of patients is an interesting subject, and it is one that each man must approach from his own standpoint, but I want you to carry home with you this thought, "What is the function of pain?" Vastly more important it is to appreciate the fact that these nerves are put in the dentine as danger signals, and that whenever we are giving our patients pain, except in extreme cases, it is our warning that we are treading on dangerous ground, and that we must look out. A paper on the Beneficence of Pain would be one of far more scientific value and use to the Association than the attempt at controlling patients. We may do a little, but that little depends upon the force of character of the operator and the material upon which he is at work.

Dr. Wm. C. Gillespie: I think that I have had two very high compliments to-night,—one by Dr. Hines, in attesting to the fact that I was absolutely an honest man, didn't try to skip anything, was going to put it all down; and another one was Dr. Morgan's closing remark.

I appreciate that very much, because I do not have any trouble with my patients at all. I get the results I have been telling you about. There is no attempt made in my paper, nor any intention, to say that I bulldoze my patient into keeping absolutely quiet; but the whole business is to control him in a common-sense way, by the use and by forcing the suggestion into his mind, mastering his will, if you please. By looking him squarely in the eye, and concentrating his mind so that I can impress it. Now if he is sitting up in his chair, looking all around at this object and that, or out of the window, he is paying no more attention to you than a jay bird, so far as impression goes. He may hear you, but you don't get any results from it, but tilt him back—just do what I say, tilt the chair back, I usually put my hand on the head-rest, or something of that sort, and looking right down at him, speak quietly, in an even tone of voice, controlling myself,—as I said in the paper. The dentist must control himself—talk to his patient intelligently and firmly, and you will get the results, just as sure as you live.

Pain has its use, exactly as Dr. Morgan says. If there were no pain we could not tell what we were doing. The only time I ever ask my patient, "Does that hurt you," is the time I expect it to hurt, and know that under usual conditions there should be a sensation of pain, and if he is so stoical that he does not manifest any pain at all, then I ask him, "Did that hurt you?" If they say no, then I must find out the reason, but the whole business is to stop this exaggerated manifestation of pain.

I did not say, as Dr. Jordan understood me to say, that all patients feel exactly the same pain. They vary just as widely as they vary in facial expression and everything else, as to how much pain they feel, but you can get the minimum expression of it, and you can get this exaggerated. This is what keeps us spending an hour or hour and a half trying to do something we should do in five minutes time. At the same time your patient does not have to go through the mental and physical exhaustion they had to endure when jumping and twisting and grabbing at you, and cutting up in all kinds of fashions.

Now I get probably just as nervous patients as any of you, I probably inflict just as much pain while I operate,—I am not a painless dentist, I never tell my patients that, but I get my results by making them submit to what pain there is to be inflicted without interfering with my work more than can possibly be avoided. If you handle your patients, you can get the same results.

Dr. Jordan says you can't do it in all sases. I would like to ask him if he tries to do this in all cases. If you try it, and do not get results, it is because you do not go at it in the right way. You are allowing your patient to turn and twist and he thinks you do not know whether you are hurting him unless he beats you to it. Instead of asking that fool question, "Did that hurt you?" And having to depend on that, I will tell you one sign you can go by that is just as clear an indication of the patient's feeling as the thermometer is of the temperature, and that is the corner of his eye. You don't have to ask him a question, but when you see that eye begin to squint, right then you know he is starting something. Then beat him to it. Lift your burr just for a revolution or two, and do that two or three times before he gets a chance to jump. I often have them ask me, "How did you know you were hurting me? How did you tell? Dr. So-and-so says to raise my hand. How did you tell?" I say, "You gave me a sign that was involuntary on your part. You didn't know you were doing it, but I am not going to tell you. I know exactly what you feel—how much I hurt you, and know before I hurt you I am going to do it, and I dont have to ask you about that." Right there is where you get your hold. Get it into his mind that you are going to protect him from useless pain, and he does not have to take care of that at all. Now he just knows that you know, somehow or other, but if you ever tell him the secrect of it, it is all off, for he will begin to bat his eye as fast as a man I heard about—but I won't tell you about it.

I appreciate your listening to the paper, I know it was crudely expressed, as it was done hurriedly.

